# Serum Magnesium Status and Its Association With Glycemic Control in Adults With Type 2 Diabetes: A Cross-Sectional Study From a Tertiary Care Center

**DOI:** 10.7759/cureus.108056

**Published:** 2026-04-30

**Authors:** Barani Sri, Sridurga Mattyvanan, Rohit S, Divya L C

**Affiliations:** 1 Biochemistry, Vinayaka Mission's Medical College and Hospital, Vinayaka Mission's Research Foundation (Deemed to be University), Karaikal, IND; 2 Biochemistry, Ramkrishna Medical College Hospital and Research Centre, Bhopal, IND

**Keywords:** electrolytes, fasting blood glucose, glycemic control, hba1c, hypomagnesemia, postprandial glucose, serum magnesium, type 2 diabetes mellitus

## Abstract

Magnesium is a crucial component for insulin production and glucose metabolism, and diminished serum magnesium levels are commonly noted in type 2 diabetes mellitus (T2DM). Despite this relevance, magnesium assessment is not frequently conducted in Indian clinical settings. This study sought to measure serum magnesium concentrations in persons with T2DM and examine their correlation with conventional glycemic indicators.

Methods

A cross-sectional analytical study was performed with 55 patients with T2DM at a tertiary care hospital. Demographic information, fasting blood sugar (FBS), postprandial blood sugar (PPBS), glycated hemoglobin (HbA1c), and serum magnesium concentrations were documented. Magnesium was quantified by spectrophotometry. Group differences were analyzed using Mann-Whitney and Kruskal-Wallis tests, correlations were evaluated with Pearson's coefficients, and linear regression was employed for predictive analysis. Hypomagnesemia was characterized by serum magnesium levels below 1.70 mg/dL.

Results

The mean serum magnesium level was 1.95 ± 0.33 mg/dL, and 12 patients (21.8%) had hypomagnesemia. Magnesium did not differ by gender (p = 0.214) or age group (p = 0.287). Magnesium levels varied significantly across HbA1c categories (p = 0.0064). Serum magnesium showed significant inverse correlations with HbA1c (r = -0.41), FBS (r = -0.28), and PPBS (r = -0.34). Patients with hypomagnesemia had higher HbA1c (9.72 ± 2.85%) than those with normal magnesium (7.65 ± 1.94%; p = 0.011). Regression analysis showed no predictive ability.

Conclusion

Hypomagnesemia is common in T2DM and is strongly associated with poorer glycemic control. Routine magnesium assessment may help identify individuals at higher metabolic risk.

## Introduction

Type 2 diabetes mellitus (T2DM) is a major metabolic disorder marked by chronic hyperglycemia and a progressive risk of microvascular and macrovascular complications. Achieving recommended HbA1c targets remains paramount for preventing long-term adverse outcomes. Yet, many individuals struggle to maintain optimal glycemic control [1]. Over the last few years, increasing attention has been given to micronutrient disturbances that may influence metabolic regulation, particularly serum magnesium deficiency.

Magnesium is an essential intracellular cation necessary for insulin secretion, activation of insulin receptors, and subsequent signaling cascades related to glucose transport and carbohydrate metabolism [2-4]. Reduced serum magnesium concentrations have been extensively documented in T2DM patients, and are thought to arise from increased urinary loss, nutritional deficiency, and disrupted homeostasis [5]. Numerous investigations have shown that hypomagnesemia is associated with increased fasting glucose, elevated HbA1c, diminished insulin sensitivity, and an increased incidence of chronic complications [6-10]. Recent clinical data consistently indicate an inverse relationship between magnesium levels and glycemic indices across various groups [11].

Recent evidence continues to substantiate the metabolic significance of magnesium. Despite these reported associations, serum magnesium measurement is not routinely performed in many outpatient diabetes clinics, especially in resource-limited settings. Understanding its pattern in real-world patients may help identify individuals who are at heightened metabolic risk.

Objective

The objective of this article was to assess serum magnesium levels in adults with T2DM attending a tertiary care hospital and to examine the relationship between magnesium concentrations and standard glycemic markers, including fasting blood sugar (FBS), postprandial blood sugar (PPBS), and HbA1c.

## Materials and methods

Study design and setting

This cross-sectional analytical investigation was undertaken in the Department of Biochemistry at Vinayaka Mission's Medical College and Hospital (VMMCH), Karaikal. Institutional Ethics Committee permission was acquired before commencing the study (IEC permission No.: VMMC/IEC/2025/MAR/05; dated 07/03/2025). The investigation followed conventional ethical guidelines and laboratory protocols as described in the institutional methodological manuals.

Study population

The study population consisted of a cohort of individuals diagnosed with T2DM attending the medicine outpatient department. Participants were eligible if they were aged above 35 years and provided written informed consent. Individuals were excluded if they had other forms of diabetes, chronic renal, hepatic, or cardiac diseases, carcinomas, thyroid abnormalities, or any other endocrine conditions known to impair magnesium homeostasis. Patients who did not consent to participate were also excluded. A total of 55 patients satisfying the inclusion and exclusion criteria were consecutively enrolled over the research period.

Ethical considerations

Informed consent was gained from all participants after discussing the study purpose, procedures, risks, and benefits in their local language. The study was conducted following the standards of the Declaration of Helsinki. Confidentiality of participant data was maintained throughout, and hazards related to venepuncture were minor and addressed as per conventional institutional protocols.

Data collection

Demographic and clinical characteristics, including age and sex, were gathered at enrollment. Biochemical markers such as fasting blood sugar (FBS), postprandial blood sugar (PPBS), glycated hemoglobin (HbA1c), and serum magnesium were tested on the same day. Glycemic control classification was carried out based on HbA1c values for further subgroup analysis.

Sample collection and processing

Venous blood samples were collected with aseptic precautions. Approximately 2 mL of blood was taken into ethylenediaminetetraacetic acid (EDTA) tubes for HbA1c estimate, and 3 mL into plain or citrate tubes for magnesium and glucose assays. Both fasting and postprandial samples were taken for glucose measurement, as indicated in the original study protocol. Samples were centrifuged shortly after collection, and the separated serum was evaluated on the same day to minimize pre-analytical variability.

Laboratory measurements

Serum magnesium concentrations were determined using a spectrophotometric method on an automated chemistry analyzer, following the manufacturer's recommendations. HbA1c was determined using a turbidimetric immunoassay technique on the laboratory autoanalyzer. FBS and PPBS were tested using the glucose oxidase-peroxidase (GOD-POD) enzymatic technique, which constitutes part of the institution's normal biochemical testing process. All assays were performed in a NABL-compliant Biochemistry laboratory with frequent internal quality control processes.

Statistical analysis

Statistical analysis was conducted using GraphPad Prism version 9 (GraphPad Software, San Diego, California). The distribution of continuous variables was examined using the Shapiro-Wilk test. As most variables revealed non-normal distribution, non-parametric approaches were used for group comparisons. Differences between the two groups were analyzed using the Mann-Whitney U test, whereas comparisons between the three groups were reviewed using the Kruskal-Wallis test with Tukey's post-hoc analysis where applicable. Associations between serum magnesium and glycemic indicators (HbA1c, FBS, PPBS), as well as age, were evaluated using Pearson's correlation coefficients. Simple linear regression models were created to examine whether HbA1c predicted serum magnesium levels and vice versa. Statistical significance was considered as p < 0.05.

Sample size justification

For the present analysis, the final sample size of 55 participants was justified based on correlation analysis demands. To detect a moderate correlation (r = 0.35-0.40) between serum magnesium and HbA1c with 80% power and a two-sided significance threshold of 0.05, a minimum of 47-52 people is required according to standard sample size formulas for correlation analysis and G*Power 3.1 calculations. Therefore, the sample of 55 patients was sufficient to discover clinically relevant relationships between the biochemical parameters tested and is equivalent to sample sizes utilized in similar cross-sectional biochemical investigations.

## Results

Descriptive characteristics of the study population

**Figure 1:**
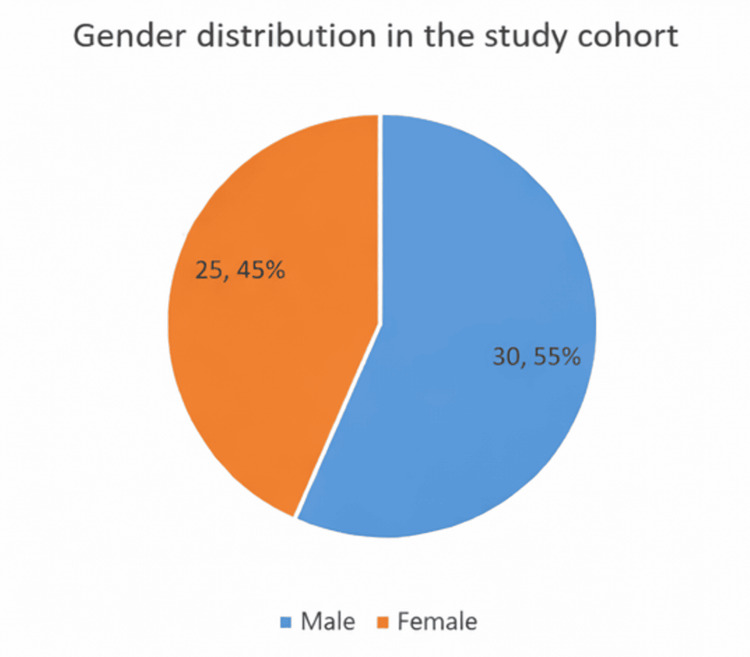
Gender distribution of the study population (n=55) The figure illustrates the proportion of male (54.5%) and female (45.5%) participants

**Figure 2:**
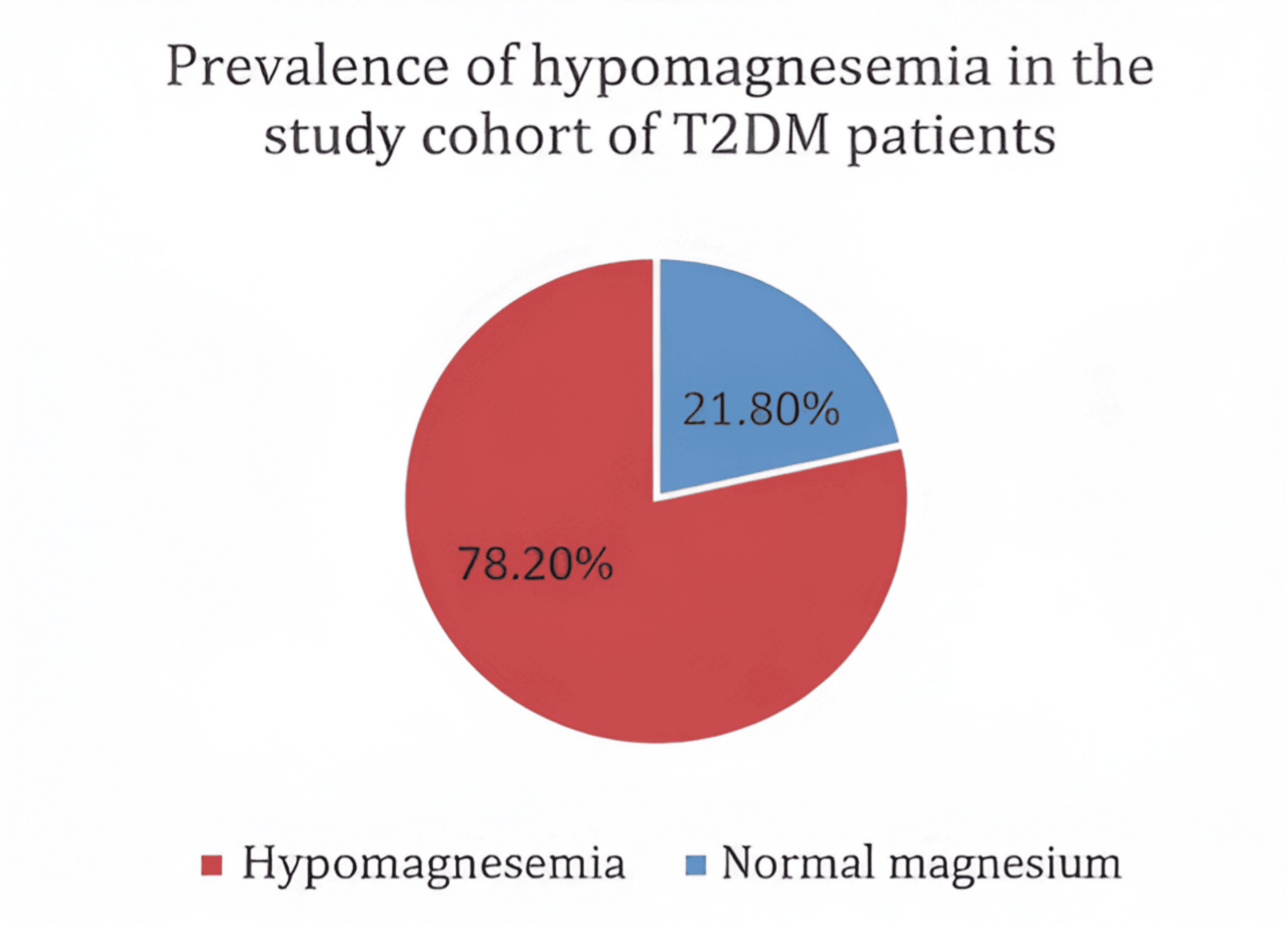
Prevalence of hypomagnesemia among study participants Hypomagnesemia (serum magnesium <1.70 mg/dL) was present in 12 out of 55 patients (21.8%)

The analysis covered a total of 55 patients. Figure 1 shows the gender distribution within the group. Of the 55 participants, 30 (54.5%) were male, and 25 (45.5%) were female. Table 1 summarizes the basic biochemical features. The average age of the study participants was 51.53 years, with a standard deviation of 12.42 years. The glycemic parameters exhibited considerable variability, as seen by a mean fasting blood sugar (FBS) of 153.7 ± 68.26 mg/dL, a postprandial blood sugar (PPBS) of 216.5 ± 87.83 mg/dL, and a mean HbA1c of 8.10 ± 2.30%. These findings suggest that most patients experienced inadequate glycemic control, as detailed in Table 1. The average serum magnesium level in the group was 1.95 ± 0.33 mg/dL. Hypomagnesemia, defined as magnesium levels below 1.7 mg/dL, was found in 12 of the 55 patients, which is 21.8% of the group. The prevalence of hypomagnesemia is depicted in Figure 2.

**Table 1:** Baseline characteristics and biochemical parameters of the study cohort (n=55) FBS - fasting blood sugar; PPBS - postprandial blood sugar

Parameter	Mean ± SD	95% CI (lower – upper)
Age (years)	51.53 ± 12.42	48.17 – 54.88
FBS (mg/dL)	153.7 ± 68.26	135.2 – 172.1
PPBS (mg/dL)	216.5 ± 87.83	192.8 – 240.3
HbA1c (%)	8.10 ± 2.30	7.48 – 8.73
Magnesium (mg/dL)	1.95 ± 0.33	1.86 – 2.04

Gender-wise comparison of serum magnesium

**Figure 3:**
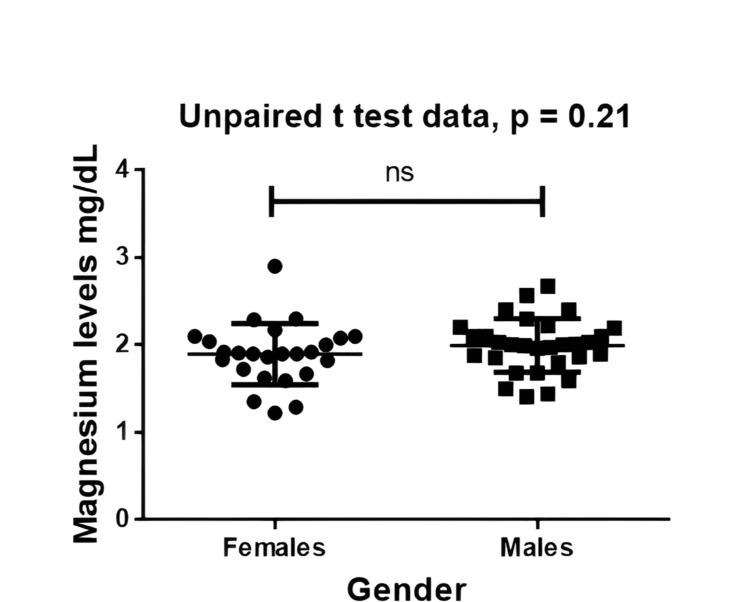
Comparison of serum magnesium levels between males and females Scatter plot comparing mean magnesium levels (males: 1.994 ± 0.307 mg/dL; females: 1.896 ± 0.350 mg/dL). Mann-Whitney U test = 301.0; p = 0.214

Serum magnesium concentrations were comparable between males and females. Female participants (n = 25) had a mean magnesium level of 1.896 ± 0.350 mg/dL, while male participants (n = 30) had a mean level of 1.994 ± 0.307 mg/dL. The difference was not statistically significant on the Mann-Whitney U test (p = 0.214), indicating that serum magnesium did not vary meaningfully by gender. Gender-wise magnesium differences are illustrated in Figure 3.

Age-wise comparison of serum magnesium

**Figure 4:**
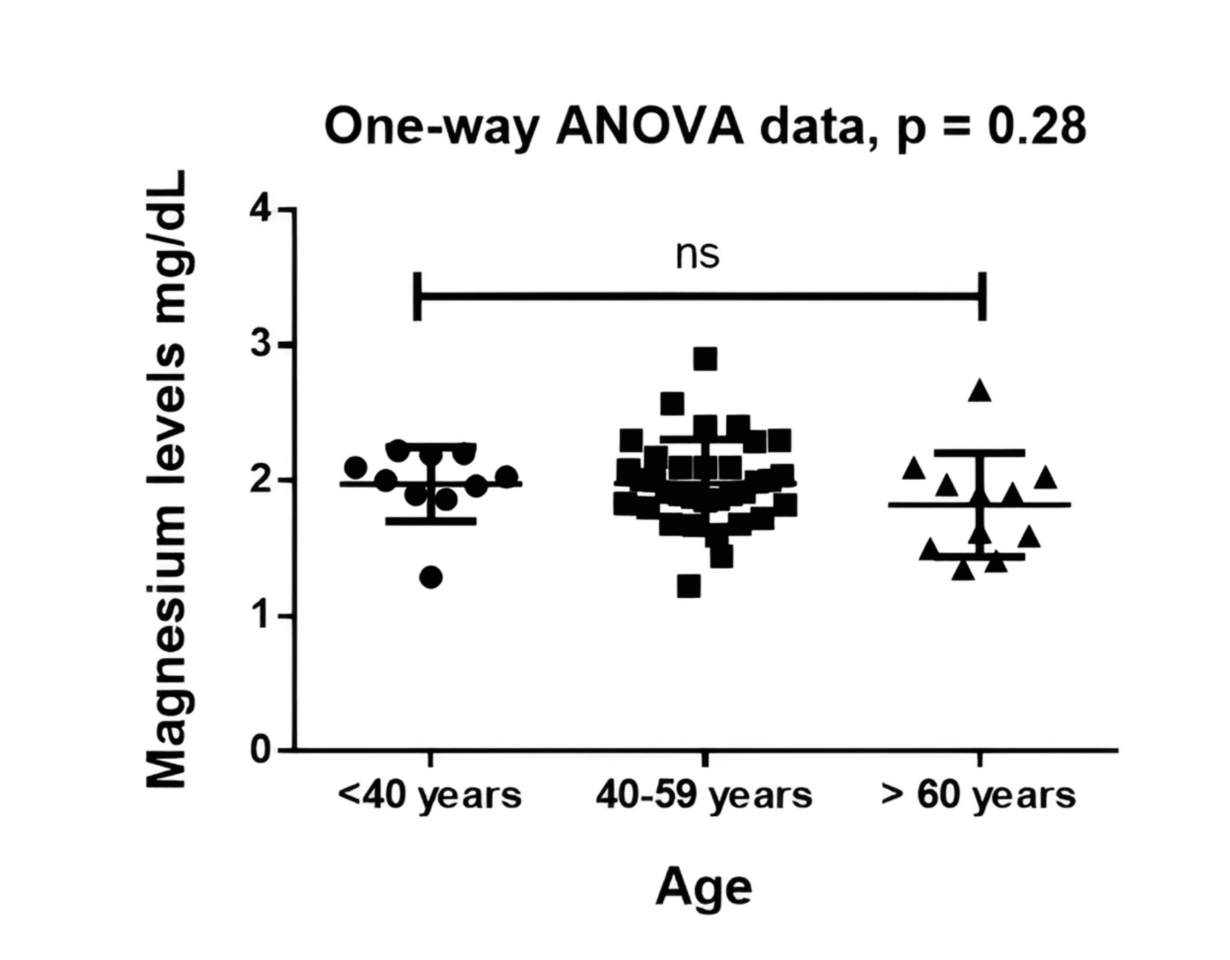
Age-wise comparison of serum magnesium levels across three age categories Magnesium levels compared in age groups <40 years (n = 10), 40–59 years (n = 34), and ≥60 years (n = 11). Kruskal–Wallis p = 0.287

Participants were grouped into three age categories: <40 years (n = 10), 40-59 years (n = 34), and ≥60 years (n = 11). The corresponding mean magnesium levels were 1.975 ± 0.272 mg/dL, 1.983 ± 0.323 mg/dL, and 1.823 ± 0.383 mg/dL, respectively. Although the ≥60-year group showed a slightly lower mean magnesium level, the overall difference across the three age categories was not statistically significant on the Kruskal-Wallis test (p = 0.287). Post-hoc Tukey comparisons confirmed no significant pairwise differences between any of the age groups. Age-group comparisons of serum magnesium are presented in Figure 4.

Magnesium levels across glycemic-control categories

Serum magnesium levels showed a significant variation across HbA1c-defined glycemic-control categories: <7% (n = 24), 7-9% (n = 17), and >9% (n = 14). The corresponding mean magnesium concentrations were 2.111 ± 0.342 mg/dL, 1.821 ± 0.243 mg/dL, and 1.829 ± 0.284 mg/dL, respectively, indicating a higher average magnesium concentration in the <7% group in comparison to the other two categories.

**Figure 5:**
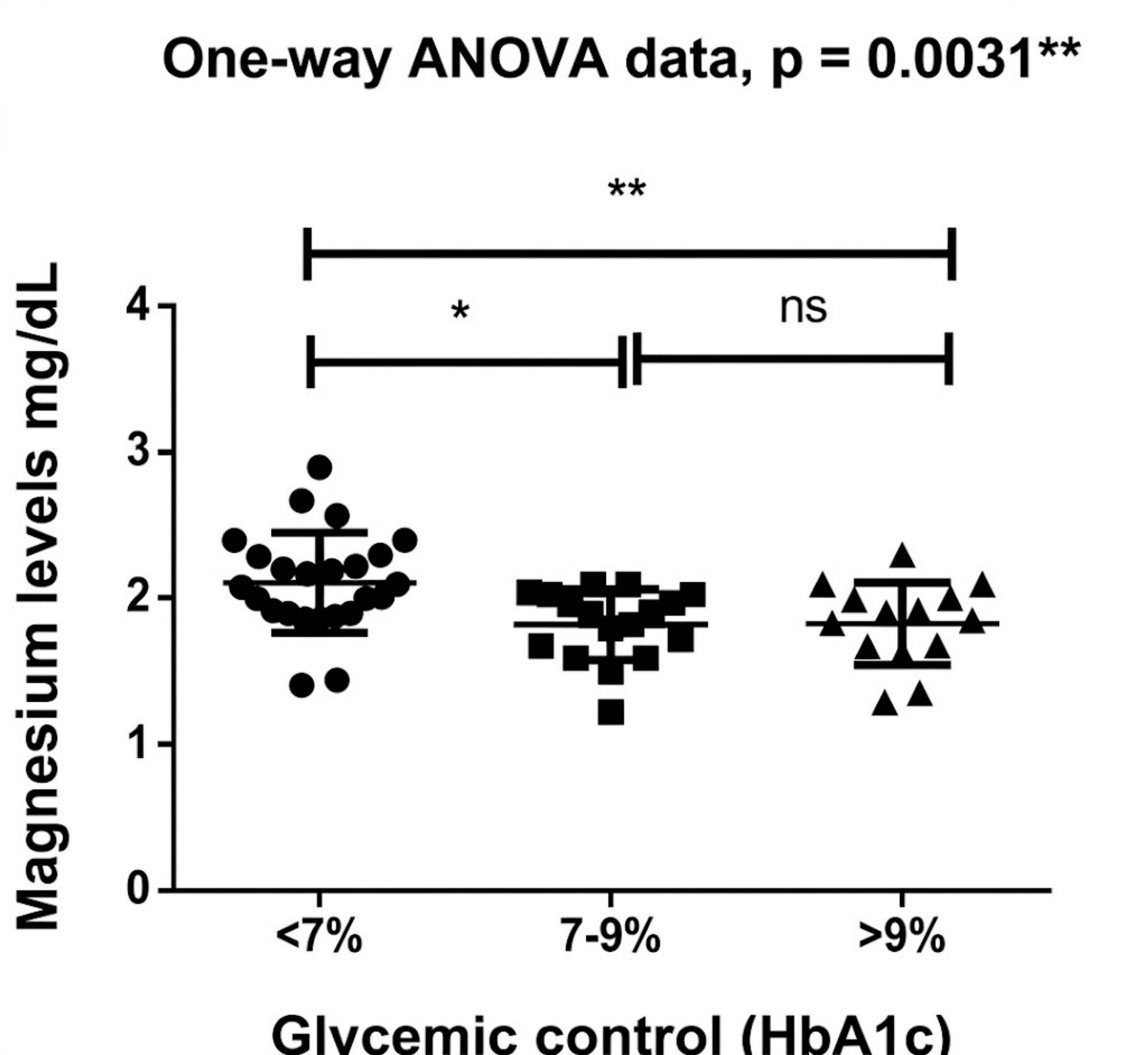
Serum magnesium levels across HbA1c-based glycemic-control categories Groups: <7% (n = 24), 7–9% (n = 17), >9% (n = 14). Kruskal–Wallis p = 0.0031 with significant pairwise differences (Tukey).

The Kruskal-Wallis test indicated a significant difference in magnesium levels across groups (p = 0.0031). Post-hoc Tukey analysis showed that individuals with HbA1c <7% had significantly higher magnesium levels compared with both the 7-9% (p < 0.01) and the >9% (p < 0.05). No significant difference was observed between the <7%, 7-9%, and >9% categories. Overall, serum magnesium levels were highest among those with mildly elevated glycemia (<7%) and comparatively lower among individuals with moderate or poor glycemic control. Magnesium levels across glycemic-control categories are plotted in Figure 5.

Correlation between serum magnesium and glycemic parameters

We used correlation analysis to examine the relationship between serum magnesium levels and glycemic variables. A statistically significant negative connection was found between HbA1c and serum magnesium (r = -0.4137, p = 0.00169). This suggests that lower magnesium levels were linked to poorer blood sugar control.

Fasting blood sugar (FBS) and postprandial blood sugar (PPBS) exhibited robust positive correlations with HbA1c, with correlation coefficients of r = 0.8059 and r = 0.8981, respectively; both p-values were less than 0.0001, thereby supporting their established biological associations.

Age showed weak connections with blood sugar levels (r ≈ 0.27-0.29; p ≈ 0.03-0.04). However, it was not substantially related to serum magnesium (r = -0.167, p = 0.222). This suggests that age did not affect the relationship between magnesium and HbA1c. Correlation coefficients between biochemical variables are summarized in Table 2.

**Table 2:** . Correlation matrix showing Pearson’s r values and corresponding p-values between key biochemical variables

Variables	FBS	PPBS	HbA1c	Magnesium	Age
FBS	—	r = 0.91 p < 0.0001	r = 0.80 p < 0.0001	r = –0.28 p = 0.037	r = 0.28 p = 0.038
PPBS	r = 0.91 p < 0.0001	—	r = 0.89 p < 0.0001	r = –0.34 p = 0.011	r = 0.27 p = 0.044
HbA1c	r = 0.80 p < 0.0001	r = 0.89 p < 0.0001	—	r = –0.41 p = 0.0016	r = 0.29 p = 0.031
Magnesium	r = –0.28 p = 0.037	r = –0.34 p = 0.011	r = –0.41 p = 0.0016	—	r = –0.16 p = 0.22
Age	r = 0.28 p = 0.038	r = 0.27 p = 0.044	r = 0.29 p = 0.031	r = –0.16 p = 0.22	—

Linear regression analysis

Simple linear regression was used to evaluate whether HbA1c could predict serum magnesium levels, but the model did not reach statistical significance. A reverse model, with HbA1c as the outcome and magnesium as the predictor, was also non-significant. Overall, while magnesium and HbA1c show an inverse association, the variability between individuals is too large for either measure to reliably predict the other through a simple linear model.

Comparison of HbA1c between hypomagnesemia and normal magnesium groups

To further investigate the clinical relevance of magnesium levels, we compared HbA1c values between people with low magnesium (<1.70 mg/dL) and those with normal magnesium levels. Hypomagnesemia was found in 12 of the 55 individuals, which is 21.8% of the total. Patients with low magnesium levels showed significantly higher HbA1c values (average 9.717 ± 2.845%, n = 12) compared to those with normal magnesium levels (average 7.653 ± 1.938%, n = 43). The difference was statistically significant, as shown by the Mann-Whitney U test (p = 0.011). This suggests that patients with lower serum magnesium levels had poorer management of their blood sugar. 

## Discussion

The present study assessed serum magnesium concentrations in adults diagnosed with T2DM, yielding three distinct observations. Firstly, serum magnesium levels were notably diminished in individuals exhibiting suboptimal glycemic control. Secondly, a negative correlation was observed between magnesium and both HbA1c and glucose measurements taken during fasting and postprandial states. Finally, hypomagnesemia was observed in nearly one in five patients (20%), underscoring that low magnesium levels are relatively common even in routine outpatient settings. These findings support current Indian and international research, which highlights magnesium's important role in the metabolic processes related to diabetes.

Several large, cross-sectional, and hospital-based studies in India have consistently shown an inverse relationship between serum magnesium levels and indicators of glycemic control. In their 2024 study in Kerala, Kumar et al. examined 230 people with diabetes. They found a significant negative relationship between magnesium levels and both HbA1c (r = -0.24) and fasting glucose (r = -0.26). In addition, those with low magnesium levels had a higher occurrence of microvascular complications [12]. Likewise, Kumar et al., in their 2026 case-control study, found that people with uncontrolled diabetes had significantly lower magnesium levels (average 1.33 mg/dL) compared to those with good diabetes control (average 2 mg/dL) [13]. Sharma et al. also found a similar negative relationship between magnesium and HbA1c (r = -0.41). This supports the idea that this metabolic connection is strong, regardless of where the research was done or how many people were in the study [14]. These observed trends support our findings and further emphasize the importance of regularly checking magnesium levels in people with T2DM.

Many studies have reported a link between low magnesium levels and complications related to diabetes. Arpaci et al. found that retinopathy and neuropathy rates were similar across different magnesium levels. However, microalbuminuria was significantly more common in people with low magnesium. Additionally, they observed a negative relationship between magnesium levels and HbA1c [15]. Moradiya et al. also found strong connections with retinopathy (OR 4.87) and nephropathy (OR 5.40). This suggests that magnesium deficiency could be an early biochemical sign of increased vascular risk [16]. Although our study didn't look at complications as we used outpatient samples, the negative relationship we found between magnesium and HbA1c in our group supports existing research. Our findings link poorer glycemic control with reduced magnesium availability.

Population studies provide further support for magnesium's role as a metabolic marker. Kao et al. studied the Atherosclerosis Risk in Communities (ARIC) cohort, which included over 12,000 adults. They found that the risk of developing diabetes increased as serum magnesium levels decreased [17]. In addition, Xu et al. studied a large group of 8010 Chinese diabetics. They found that higher magnesium levels were independently linked to lower rates of obesity and abdominal obesity. This relationship was partly explained by reduced inflammation [18]. These findings suggest that magnesium status is not just an indicator of how well blood sugar is controlled, but may also reflect a broader metabolic state, including inflammation, fat storage, and the risk of developing diabetes over time.

Additional high-quality evidence also supports these observations. A large meta-analysis of 13 prospective cohort studies by Dong et al. demonstrated a clear dose-response inverse association between magnesium intake and the future risk of developing diabetes, involving more than half a million participants followed over several years [19]. This large-scale epidemiological evidence reinforces the biological plausibility of our findings and indicates that the relationship between magnesium status and glycemic regulation extends beyond cross-sectional settings. Our study adds to this body of literature by showing that, even in a real-world outpatient population, lower serum magnesium levels accompany higher glycemic indices, with hypomagnesemia detected in nearly one in five adults with T2DM.

These findings should be regarded with some caution. The study, conducted at a single center with a modest cohort of participants, didn't consider things like dietary magnesium, and specific kidney functions were not assessed. The cross-sectional design also limits our capacity to establish the direction of the relationships we observe. Nevertheless, using standard laboratory tests and proper statistical methodologies provides a practical, real-world understanding of how magnesium relates to glycemic control in everyday diabetic care. Future studies, with larger prospective cohorts and eventually intervention trials, will help clarify the relationship between changes in magnesium levels and glycemic control over time.

## Conclusions

In conclusion, this study reinforces the consistent, inverse relationship between serum magnesium levels and measures of blood sugar control in people with T2DM. Hypomagnesemia appears to be widespread and clinically relevant, even in typical outpatient settings. Monitoring magnesium levels could give healthcare professionals a readily available indicator of metabolic stability, allowing for early interventions to address imbalances. This would complement existing methods for managing diabetes
